# Solution of Nitro-Sulphate of Iron

**Published:** 1869-01-15

**Authors:** Z. C. McElroy

**Affiliations:** Zanesville, Ohio, President Muskingum County, Ohio, Medical Society


### *Solution of Nitro-Sulphate of Iron.*

BY Z. C. MC ELROY, M. D., ZANESVILLE, OHIO, PRESIDENT MUSKINGUM COUNTY, OHIO, MEDICAL SOCIETY.

The formidable list of preparations of iron in our national pharmacopœia, would seem to forbid the idea that the whole ground was not most fully covered; and that any addition must have very conclusive reasons for its recognition by the profession. Notwithstanding their number, however, the manufacture of preparations not recognized by the pharmacopœia, on a very large scale, by various commercial houses, is a well known fact; as is, also, another fact, to-wit: that they are largely used by the professions. That they supply a want may as well be admitted as a still further fact. No doubt various causes contribute to these ends; but a main one certainly is, their pharmaceutical elegance, and agreeable sensible properties. The elixirs of calisaya and iron, and with bismuth, must be known to all who take and read medical periodicals; and any defect in their mode of making them known, would be compensated for by the active, polite, and vigilant commercial agents of the houses engaged in their manufacture.

Recognizing in iron a therapeutic agent of the very highest importance, it has often seemed to me that some form, which could be readily put up extemporaneously, having the elegance and agreeable sensible properties of the popular elixirs, was still a desideratum to the general practitioner, if only for the sake of knowing precisely what he was prescribing.

A personal and professional friend of the writer, Dr. J. R. Black, of Newark, Ohio, communicated to the *Cincinnati Lancet and Observer*, for March, 1868, a formula for the preparation of what he continued to call, probably because it was the name by which he received it, "Liquid oxysulphate of iron." It is as follows:

℞. Ferri Sulph: ʒ ij:

Acid Nitric: j ʒ iij;

Aqua Distill: ℥ iss;

Rub the sulphate with the acid in a mortar, gradually add the water after the sulphate is all dissolved, and filter through paper. Dose from 7 to 12 drops in water or quassia infusion.

Though this is given verbatim, as reported by Dr. Black, a very slight examination suffices to discover its want of scientific exactness. There is no quantity indicated at the close of the process. No statement as to the quality of either the sulphate of iron or nitric acid. It seems to me that these should be *pure*, not *commercial*, and that at the end of the process there should be one and one-half ounces of the solution; even if it falls short, distilled water should be passed through the filter until that quantity is obtained. If in excess, evaporate on sand bath.

The writer is not quite chemist enough to state precisely what changes occur on the addition of the acid to the sulphate. Large volumes of nitric oxide, becoming, probably, hyponitric acid by contact with the atmosphere are liberated. It is probably a tersulphate, with considerable free nitric acid in the resulting solution. Its color is very different from the tersulphate of the pharmacopœia. So that it must be that and something else; and that something else, most likely, free acid and water. After passing through the filter the solution is a very beautiful pale amber color, and seems to be quite stable, as a sample was examined this very morning at an apothecary's, more than a month old, which showed neither precipitate or change in any way. Its chemical formula is probably that for per sulphate of iron, plus nitric acid and water, and might be written in chemical symbols as follows: $Fe_2\,O_3 . 3\,S\,O_3 + N\,O_3 + H\,O$. It mixes freely in all proportions with water, spirits and simple syrup, without altering its beautiful color. Its incompatibles are tannin, gallic and malic acids. The point of most practical importance to the general practitioner is, that here is an acid salt of iron in solution, which, in extemporaneous prescriptions, without incompatibles, rivals in pharmaceutical elegance, and pleasant taste any of the commercial elixirs of calisaya, iron and bismuth. Sulphate of quinia may be added in any proportion, instantly dissolving, thus forming a suitable elixir of calisaya and iron, without change of color, but acquiring the bitter taste of quinia in proportion to the quantity added.

Now suppose an elegant acid iron preparation be desired, let the following formula serve as a basis, for an indefinite number of variations:

| | | |
|---|---|---|
| ℞. | Cinnamon water, | f ʒ v; |
| | Simple syrup, | f ℥ ij; |
| | Diluted alcohol, | ℥ j; |
| | Sol. Nitro-sulphate of Iron, | ʒ iij; |

Dose, a teaspoonful for an adult.

Brandy and whisky both discolor the mixture, as do most of the aromatic tinctures. The aroma must come from the volatile oils, either through the alcohol, or pure white spirits or water. To preserve its elegance it is only needful to remember that nothing containing tannin, etc., can be added without darkening the color, though where this is not objectionable, the most valuable part of the sensible properties, to-wit: pleasant taste is preserved. It is a gratifying ing fact that this preparation is seldom indicated in connection with its incompatibles. The indications for acid preparations of iron are usually sharply defined. Broadly viewing the *modus oporandi* of organic life in the human body, it is seen that in the construction of its various tissues, textures and organs, out of other organic material, the acid elements predominate; without acids there can be no digestion in the human stomach, for it is in truth an acid alembic The mission of alkalies, and the salts they form with most acids is to arrest construction, and to break down and remove effete matter from the system.

For diseases characterized by great waste or destruction of tissue, the mixture of the chloride of iron, an acid preparation of the pharmacopœia, has been found a very valuable remedy. Why? Because it is an acid preparation of iron, and acids, better than any other preparation, stay the processes of destruction and promote those of repair. That is just why large doses of the common tincture of iron are is not so urgent, as in anæmic and chlerotic conditions, so efficacious in erysipelas and diphtheria. When the waste iron in combination with organic acids; or pure, as in the ferrum reductum; or neutral, as in the precipitated carbonate, are very appropriate, and very highly useful, but will disappoint if relied on to stay rapid wasting. These generalizations being conceded, and they are original with the writer, it necessarily follows that in children debilitated by bowel complaint, the solution of the nitro-sulphate of iron fulfils all the indications needful to recovery with elegance and agreeable pharmacy. So, too, it would be appropriate in adults debilitated by excessive discharges from the skin, bowels or lungs; no better, perhaps, than the common Tinct. Ferr. Chlor., but far more elegant and pleasant to the taste, and, therefore, in many cases, more likely to be taken, which, are after all very weighty considerations in highly civilized life, whether in city or country. It is considered an imperative duty on myself to present medicines in their most agreeable forms to those who seek my professional counsels.

It can readily be seen, then, that while the number of preparations of iron is very large, this acid sulphate fulfills certain indications, better than any one of the long list, and from its cheapness, elegance and efficiency, has its own place among them.

Before me stand some "physicians' sample packages," placed on my table by a very polite agent of the manufacturers, of what they call "Elixir of Calisaya Bark and Iron," and the same with "bismuth," whose pharmacy is all that could be desired. Each package has a printed formula of its contents. Some of the ingredients, as for instance, brandy and whisky, contain tannin, and yet the solutions are beautifully clear and pleasant to the taste. Now, these formulas cannot by any possibility represent truthfully the contents of these "sample packages," for, if brandy or whisky are added to any of the pale solutions of iron, the color is quickly changed to some more or less dark tint. And as these formulas are clearly not indicative of the contents of these packages, none but those in the secret of their manufacture can tell precisely what they do contain, save that some aromatic spirits, sugar and iron are present; but of how much of each, and the mode of preparation, we are left entirely in the dark. Not so, however, withthe extemporaneous preparations of the acid sulphate, for apothecaries will put up what the physician's prescription calls for, and hence, when thus written for, the physician has a definite knowledge of what the patient is to take.

After trial, others, like myself, will thank Dr. Black for communicating the formula to the profession, even though it is wanting in scientific exactness. To supply, to some extent, at least, this defect is the object of this paper. It has been used by the writer a great many times, and with very satisfactory results ; for the reason that patients, both adults and children, will take pleasant medicine regularly, when they are very apt to forget it or postpone it, if it is unpleasant to the taste, perhaps, more particularly in chronic cases.

